# Investigator-Driven Randomised Controlled Trial of Cefiderocol versus Standard Therapy for Healthcare-Associated and Hospital-Acquired Gram-negative Bloodstream Infection: Study protocol (the GAME CHANGER trial): study protocol for an open-label, randomised controlled trial

**DOI:** 10.1186/s13063-021-05870-w

**Published:** 2021-12-07

**Authors:** Hugh Wright, Patrick N. A. Harris, Mark D. Chatfield, David Lye, Andrew Henderson, Tiffany Harris-Brown, Anna Donaldson, David L. Paterson

**Affiliations:** 1 Centre for Clinical Research, Faculty of Medicine, University of Queensland, Royal Brisbane and Women’s Hospital Campus, Brisbane, Queensland Australia; 2 Department of Infectious Diseases, Royal Brisbane and Women’s Hospital, Brisbane, Queensland Australia; 3 Department of Microbiology, Pathology Queensland, Royal Brisbane and Women’s Hospital, Brisbane, Queensland Australia; 4 Infectious diseases research and Training Office, National Centre for Infectious Diseases, Singapore, Singapore; 5 Department of Infectious Diseases, Tan Tock Seng Hospital, Singapore, Singapore; 6 Yong Loo School of Medicine, Singapore, Singapore; 7 Lee Chian School of Medicine, Singapore, Singapore; 8 Department of Infectious Diseases, Princess Alexandra Hospital, Brisbane, Queensland Australia

**Keywords:** Extended-spectrum beta-lactamase, Carbapenem, Cefiderocol, Clinical trial, Multi-drug resistance

## Abstract

**Background:**

Increasing rates of antibiotic resistance in Gram-negative organisms due to the presence of extended-spectrum beta-lactamases (ESBL), hyperproduction of AmpC enzymes, carbapenemases and other mechanisms of resistance are identified in common hospital- and healthcare-associated pathogens including Enterobacteriaceae, *Pseudomonas aeruginosa* and *Acinetobacter baumannii*.

Cefiderocol is a novel siderophore cephalosporin antibiotic with a catechol moiety on the 3-position side chain. Cefiderocol has been shown to be potent in vitro against a broad range of Gram-negative organisms, including carbapenem-resistant Enterobacteriaceae (CRE) and multi-drug-resistant (MDR) *P. aeruginosa* and *A. baumannii*. Recent clinical data has shown cefiderocol to be effective in the setting of complicated urinary tract infections and nosocomial pneumonia, but it has not yet been studied as treatment of bloodstream infection.

**Methods:**

This study will use a multicentre, open-label non-inferiority trial design comparing cefiderocol and standard of care antibiotics. Eligible participants will be adult inpatients who are diagnosed with a bloodstream infection with a Gram-negative organism on the basis of a positive blood culture result where the acquisition meets the definition for healthcare-associated or hospital-acquired. It will compare cefiderocol with the current standard of care (SOC) antibiotic regimen according to the patient’s treating clinician. Eligible participants will be randomised 1:1 to cefiderocol or SOC and receive 5–14 days of antibiotic therapy. Trial recruitment will occur in at least 20 sites in ten countries (Australia, Malaysia, Singapore, Thailand, Turkey and Greece). The sample size has been derived from an estimated 14 day, all-cause mortality rate of 10% in the control group, and a non-inferiority margin of 10% difference in the two groups. A minimum of 284 patients are required in total to achieve 80% power with a two-sided alpha level of 0.05. Data describing demographic information, risk factors, concomitant antibiotics, illness scores, microbiology, multidrug-resistant organism screening, discharge and mortality will be collected.

**Discussion:**

With increasing antimicrobial resistance, there is a need for the development of new antibiotics with broad activity against Gram-negative pathogens such as cefiderocol. By selecting a population at risk for multi-drug-resistant pathogens and commencing study treatment early in the clinical illness (within 48 h of index blood culture) the trial hopes to provide guidance to clinicians of the efficacy of this novel agent.

**Trial registration:**

The GAME CHANGER trial is registered under the US National Institute of Health ClinicalTrials.gov register, reference number NCT03869437. Registered on March 11, 2019.

## Administrative information

Note: the numbers in curly brackets in this protocol refer to SPIRIT checklist item numbers. The order of the items has been modified to group similar items (see http://www.equator-network.org/reporting-guidelines/spirit-2013-statement-defining-standard-protocol-items-for-clinical-trials/).

**Table:** 

Title {1}	Investigator Driven Randomized Controlled Trial of Cefiderocol versus Standard Therapy for Healthcare Associated and Hospital Acquired Gram-negative Blood Stream Infection: Study protocol (the GAME CHANGER trial)
Trial registration {2a and 2b}.	ClinicalTrials.gov identifier: NCT03869437
Protocol version {3}	Version 7, 20th May, 2020
Funding {4}	The trial is funded by Shionogi Pharmaceutical through their Investigator Initiated Studies program Limited investigator Initiated Funding.
Author details {5a}	Hugh Wright^,1,2^, Patrick NA Harris^1,3^, Mark D Chatfield^1^, , David L Paterson^1,2^ ^1^Centre for Clinical Research, Faculty of Medicine, University of Queensland, Royal Brisbane and Women’s Hospital Campus, Brisbane, Queensland, Australia ^2^Department of Infectious Diseases, Royal Brisbane and Women’s Hospital, Brisbane, Queensland, Australia ^3^Department of Microbiology, Pathology Queensland, Royal Brisbane and Women’s Hospital, Brisbane, Queensland, Australia
Name and contact information for the trial sponsor {5b}	University of Queensland St Lucia Queensland Australia, 4072
Role of sponsor {5c}	The study sponsor is the University of Queensland. The Principal Investigator and the research team (authors) are responsible for the study design, collection, management, analysis, and interpretation of data and writing of the report or publication. The funder has no role in the study conduct, analysis and interpretation of the findings, and dissemination of the results.

## Introduction

### Background and rationale {6a}

Infections with antibiotic-resistant bacteria cause a significant burden of disease worldwide. Bloodstream infections may arise from a variety of sources, are commonly encountered in clinical practice, and are associated with significant morbidity and mortality. Antibiotics that have activity against a broad spectrum of pathogens are commonly suggested in treatment guidelines to adequately cover bloodstream infections. Increasing rates of resistance to antibiotics commonly used for bloodstream infection are problematic and may lead to initial empiric therapy not having activity against the pathogen isolated [1]. In patients with bloodstream infections and sepsis, delay until the receipt of effective therapy is associated with an increase in mortality [2, 3].

Increasing rates of antibiotic resistance in Gram-negative organisms due to the presence of extended-spectrum beta-lactamases (ESBL), hyperproduction of AmpC enzymes, carbapenemases and other mechanisms of resistance are identified in common hospital- and healthcare-associated pathogens including Enterobacteriaceae, *Pseudomonas aeruginosa* and *Acinetobacter baumannii* [4].

Cefiderocol (previously S-649266) is a novel siderophore cephalosporin antibiotic with a catechol moiety on the 3-position side chain. Cefiderocol has been shown to be potent in vitro against a broad range of Gram-negative organisms, including carbapenem-resistant Enterobacteriaceae (CRE) and multi-drug-resistant (MDR) *P. aeruginosa* and *A. baumannii* [5, 6]. Recent clinical data has shown cefiderocol to be effective in the setting of complicated urinary tract infections and nosocomial pneumonia [7, 8]. In a descriptive study of patients with severe diseases caused by carbapenem non-susceptible pathogens [9], more deaths were observed in participants who received cefiderocol, though this did not meet statistical significance. As such, further data is needed on the role of cefiderocol in the treatment of bloodstream infections. Given the broad spectrum of activity against Gram-negative organisms, including those with resistant phenotypes, cefiderocol may be an ideal agent for use in the setting of bloodstream infections acquired in the hospital or healthcare setting but to date, no clinical trial has examined this.

Our hypothesis is that cefiderocol is non-inferior to the current standard of care treatment for Gram-negative bloodstream infection that is healthcare-associated or hospital-acquired. Cefiderocol is active against a broad spectrum of Gram-negative pathogens including multi-drug-resistant isolates and may be potentially advantageous to use as an empiric treatment where the risk of multi-drug resistance is high.

### Objectives {7}

#### Primary objective

To compare the 14-day mortality from the day of randomisation of each regimen (cefiderocol versus standard of care therapy)

#### Secondary objectives

1. To compare mortality post bloodstream infection of each regimen at longer time points (30 and 90 days)
2. To compare clinical and microbiologic success of each regimen
3. To compare the functional outcome of patients treated with each regimen
4. To compare the rates of relapse of bloodstream infection (microbiological failure) with each regimen
5. To compare lengths of hospital (acute) and ICU stay with each regimen
6. To compare the number of treatment emergent serious adverse events with each regimen
7. To compare rates of *Clostridium difficile* infection *(*CDI) with each regimen
8. To compare rates of colonisation and/or infection with multi-resistant bacterial organisms (MROs) including those newly acquired

### Trial design {8}

The study is an open-label randomised, controlled non-inferiority trial design comparing two drug regimens, with a 1: 1 randomisation to cefiderocol vs. standard of care therapy for bloodstream infections caused by Gram-negative organisms that are hospital- or healthcare-associated. Commonly used agents in the standard of care regime include carbapenems, piperacillin-tazobactam, cefepime with or without adjuvant aminoglycoside therapy; other regimens including ceftazidime-avibactam or other beta-lactam/beta-lactamase inhibitor combinations, polymyxins, tigecycline and in some regions fosfomycin are allowable as defined by the treating physician if these antibiotics are appropriate because of resistance or clinical factors.

## Methods: Participants, interventions and outcomes

### Study setting {9}

The study is an international, multi-centre hospital-based study with sites in Australia, Thailand, Malaysia, Singapore, Turkey and Greece. A list of study sites can be found at ClinicalTrials.gov study identifier: NCT03869437.

### Eligibility criteria {10}

#### Inclusion criteria

1. Bloodstream infection with a Gram-negative bacilli from at least one blood culture draw. Enrolment will be based on the Gram stain from blood culture bottles flagged positively by an automated system used to incubate blood cultures and detect bacterial growth (e.g. Bactec or BacTAlert).
2. The bloodstream infection fulfils the criteria as a hospital-acquired or healthcare-associated infection as per the following definitions
  - Hospital-acquired – Bloodstream infection occurring greater than 48 h after hospital admission, assessed as symptoms or signs of infection not present at the time of hospital admission
  - Healthcare-associated – Bloodstream infection present at admission to hospital or within 48 h of admission in patients that fulfil any of the following criteria:
    i. Patient has an intravascular catheter/line that is the source of infection
    ii. Attended a hospital or haemodialysis clinic or received intravenous chemotherapy in the previous 30 days
    iii. Were hospitalised in an acute care hospital for two or more days in the previous 90 days
    iv. Resided in a nursing home or long-term care facility
    v. Received intravenous antibiotic therapy at home, wound care or specialised nursing care through a healthcare agency, family or friends; or had self-administered intravenous antibiotic medical therapy in the 30 days before the infection
3. No more than 48 h has elapsed since the positive blood culture collection.
4. Patient is aged 18 years and over (21 in Singapore)
5. The patient or approved proxy is able to provide informed consent.

#### Exclusion criteria

1. Refractory shock or comorbid condition such that patient not expected to survive more than 7 days as per the judgement of the treating clinician.
2. Patient with a history of moderate to severe hypersensitivity reaction to a cephalosporin
3. Patient with Gram-positive bacteraemia including a significant Gram-positive pathogen (a Gram-positive skin contaminant in one set of blood cultures may not be regarded as significant).
4. Where the bloodstream infection is thought to be related to a vascular catheter and the catheter is unable to be removed.
5. Treatment is not with the intent to cure the infection (that is, palliative care is an exclusion).
6. Known pregnancy or breast-feeding.
7. Patient is receiving peritoneal dialysis
8. Patients previously randomised in this trial

### Who will take informed consent? {26a}

Potential participants will be identified on the basis of the microbiology laboratory detecting a positive blood culture with Gram stain showing Gram-negative bacteria. The site investigator or their delegate will then be notified. The site investigator or their delegate will approach the doctors of the treating team and ask permission to approach the patient or their surrogate decision-maker. When permission is obtained the site investigator or delegate will discuss the risks, benefits and nature of the trial. The participant will be given the opportunity to ask any relevant questions and be given a copy of the Human Research Ethics Committee/Institutional Review Board approved informed consent form. The right of a participant to refuse participation without giving reasons will be respected.

Alternative methods for supporting the informed consent process will be employed in the event of inability to read and write, require translation or have cognitive impairment. Approved substitute decision-maker (SDM) consent processes will be provided. The participant/SDM are free to withdraw from the trial at any time without giving reasons and without prejudicing the participant’s further treatment.

All site investigators and delegates will be trained and competent to participate according to the ethically approved protocol, principles of Good Clinical Practice (GCP) and Declaration of Helsinki.

### Additional consent provisions for collection and use of participant data and biological specimens {26b}

The informed consent process includes notification and discussion with participants on an extra laboratory testing that will be required as part of study participation.

## Interventions

### Explanation for the choice of comparators {6b}

The trial is pragmatic in nature, allowing the comparator to be the standard therapy that the treating clinician would use in routine clinical practice as per the presenting clinical syndrome. Agents in common use include carbapenems, extended-spectrum beta-lactams such as piperacillin-tazobactam or cefepime. Aminoglycosides are frequently used as empiric adjuvant therapy. Other agents that may be used include tigecycline, ceftazidime-avibactam and polymixins depending on local practice and the rates of multi-drug resistance seen locally at the study site.

### Intervention description {11a}

Cefiderocol will be provided by Shionogi & Company, Ltd, (Osaka Japan) and supplied to sites through an identified certified distributor. Cefiderocol will be labelled as an investigational product and only used for patients enrolled and randomised in this study.

Participants will receive cefiderocol 2 g administered intravenously over 3 h, every 8 h with adjustment made based on an assessment of renal function.

Dose adjustment for renal impairment will be made according to the criteria below. Patients receiving peritoneal dialysis are excluded from the trial. Blinding will not be performed.

**Table:** 

Renal function	Dose (grams)	Frequency (hours)	Infusion time (hours)
Normal renal function (CrCL 90 to < 120 mL/min)	2 g	Every 8 h	3 h
Mild renal impairment (CrCL 60 to < 90 mL/min)	2 g	Every 8 h	3 h
Moderate renal impairment (CrCL 30 to < 60 mL/min)	1.5 g	Every 8 h	3 h
Severe renal impairment (CrCL 15 to < 30 mL/min)	1 g	Every 8 h	3 h
ESRD (CrCL < 15 mL/min)	0.75 g	Every 12 h	3 h
Patient with intermittent Had	0.75 g	Every 12 h	3 h
Patient with CVVH	1 g	Every 12 h	3 h
Patient with CVVHD or CVVHDF	1.5 g	Every 12 h	3 h

In the standard of care arm, treatment may include one, two or three antibiotics with activity against Gram-negative bacteria. Additional antibiotics may be given to treat Gram-positive or anaerobic pathogens.

Dosage, frequency and administration will be dictated by the treating physician.

In the cefiderocol arm, the use of a second Gram-negative antibiotic (adjuvant therapy) that has activity against Gram-negative organisms will be allowed in the first 72 h post-randomisation until susceptibility of cefiderocol is confirmed.

Given cefiderocol has limited activity against anaerobic Gram-negative organisms metronidazole will be permitted to be added in the setting where the bloodstream infection is considered a result of polymicrobial intra-abdominal infection.

Treatment may include one, two or three antibiotics with activity against Gram-negative bacteria. Additional antibiotics may be given to treat Gram-positive or anaerobic pathogens.

### Criteria for discontinuing or modifying allocated interventions {11b}

Duration of study drug administration will be at the discretion of the treating clinician and consent of the participant for a minimum of 5 days to a maximum of 14 days.

In the event of microbiological failure of the treatment given, defined as ongoing growth of the index isolate in blood cultures on day seven or later post-randomisation, “rescue” therapy will be allowed at the discretion of the treating clinician. This includes the use of cefiderocol in the SOC arm or the addition of other agents with activity against Gram-negative pathogens in the cefiderocol arm. Patients who receive “rescue” therapy will be assessed as treatment failures in the primary analysis.

### Strategies to improve adherence to interventions {11c}

Participants will be in-patients for intervention, therefore control of drug dose will be by the treating clinical team and participant’s consent.

### Relevant concomitant care permitted or prohibited during the trial {11d}

Trimethoprim-sulfamethoxazole may be continued in patients who require *Pneumocystis* prophylaxis. For patients randomised to cefiderocol, antibiotics active against aerobic Gram-negative bacilli are not permitted after 72 h of study drug therapy.

### Provisions for post-trial care {30}

Randomised participants can receive a minimum of 5 days to a maximum of 14 days of allocated treatment. Participants may meet the criteria of withdrawal at any point post-randomisation during the treatment period. On experiencing an adverse event, completion, or early withdrawal of study treatment the treating clinician will direct any required routine clinical care. Participants will not receive payment for involvement within the study but will receive all usual cares including treatment of any adverse effects that occur.

### Outcomes {12}

The following tables describe the primary and secondary outcomes and criteria of evaluation (Tables 1 and 2).

**Table 1:** Primary objective and outcome

Objective	Outcome measure	Time point(s) of evaluation
To compare all-cause mortality of each regimen	Vital status (alive or dead)	14 days after randomisation

**Table 2:** Secondary objectives and outcomes

#	Objectives	Outcome measures	Time point(s) of evaluation
1	To compare all-cause mortality of each regimen	Vital status (alive or dead)	Day 30 and day 90
2	To compare clinical and microbiologic success of each regimen at day 14	1. Vital status (alive or dead) 2. SOFA score (ICU) or modified SOFA score (non-ICU) stable or improved 3. Microbiological cure defined as no growth in blood of index isolate on day 7 or later post randomisation (taken only if the patient is febrile ≥ 38 °C, to prevent unnecessary additional protocol-driven blood collection (afebrile patients have presumed eradication)	1. Day 14 2. Day 1 and day 14 3. Day 7 to day 14
3	To compare the functional outcome of patients treated with each regimen	Baseline and 30-day post-randomisation Functional Bacteremia Outcome Score. NB. Baseline reflects pre-admission status prior to condition meriting hospital admission.	Screening and day 30
4	To compare the rates of relapse of bloodstream infection (microbiological failure) with each regimen	Growth of the same organism as index blood culture	Post cessation of randomised treatment up to day 90
5	To compare lengths of hospital (acute) and ICU stay with each regimen	Number of days at home. ICU ± non-ICU stay defined as duration between index blood culture and 90-day post-randomisation	Cumulative up to day 90
6	To compare the number of treatment emergent serious adverse events with each regimen	Treatment emergent serious adverse events	Day 1 to the last dose plus 5 days
7	To compare rates of *Clostridium difficile* infection *(*CDI) with each regimen	Clinician diagnosed (including a positive CDI test) and treated CDI	30 days
8	To compare rates of colonisation and/or infection with multi-resistant bacterial organisms (MROs) including those newly acquired	1. New MROs detected from any clinical specimen post cessation of randomised treatment. MROs include vancomycin-resistant Enterococci (VRE), methicillin-resistant *Staphylococcus aureus* (MRSA) and multi-resistant Gram-negative organisms including carbapenem-resistant Enterobacteriaceae carbapenem-resistant *Pseudomonas aeruginosa* (CRP), carbapenem-resistant *Acinetobacter baumannii* (CRAB)	Baseline and up to day 30

### Participant timeline {13}

The participant timeline is shown in Table 3.

**Table 3:** Study time and event schedule

Treatment day	Screen	1	2	3	4	5	6	7	8–13	14	30	90
**Eligibility assessments**												
Informed consent	x											
Inclusion/exclusion criteria	x											
Medical history	x											
Demographic data	x											
Concomitant medications	x	x	x	x	x	x	x	x	x	x		
Randomisation		x										
**Laboratory tests**												
Microbiology: Blood cultures	(x)^a^			x		x^c^		(x)^b^	(x)^b^	(x)^b^		
Haematology^d^ Biochemistry^e^		x		x		x		x				
**Safety assessments**												
Daily monitoring assessment			x	x	x	x	x	x	x	x		
SOFA score assessment	x					x				x		
Vital status (alive)		x	x	x	x	x	x	x		x	x	x
Adverse events assessments		x	x	x	x	x	(x)^g^	(x)^g^	(x)^g^	(x)^g^		
**Outcome assessments**												
Functional bacteraemia outcome score		x									x	
Follow-up data collection/review										x	x	x

^a^If > 24 h has elapsed after index positive blood culture taken

^b^Blood cultures taken if patient febrile > 38 °C in last 24 h or previous days blood cultures positive

^c^Blood cultures to assess clearance if day 3 cultures positive

^d^Haematology includes haemoglobin, white cells, neutrophils, platelets

^e^Biochemistry includes electrolytes, creatinine (or eGFR), ALT, AST, ALP and total bilirubin. Note not all values are required to be captured in the electronic database, but all values should be recorded in the medical notes and may be requested by sponsor for safety assessments.

^g^Only if still on study drug (AE reporting to cease 5 days post last study dose)

### Sample size {14}

The sample size estimation has been derived from retrospective studies of bloodstream infection from Gram-negative organisms. Considering a mortality rate of 10% in the control group, and a non-inferiority margin of 10% difference in the two groups, we would need a minimum of 284 patients in total to achieve 80% power with a two-sided alpha level of 0.05. The mortality rate in the control group has been based on previous clinical trials examining Gram-negative bloodstream infections, though significant variability has been reported [10, 11]. As such, no non-inferiority margin has been judged to show a clinically relevant difference between the treatment groups and is similar to other trials examining this area [12]. As recommended by the CONSORT statement, we will report relative risk ratios as well as absolute risk differences.

### Recruitment {15}

We plan on conducting this trial over a 3-year time period. Identification of patients who have cultured Gram-negative organism from a blood culture by the microbiology department of the study sites will be communicated to the study staff who will then approach the treating clinicians. Expansion to other study sites may be considered to boost recruitment.

## Assignment of interventions: allocation

### Sequence generation {16a}

A computer-generated random sequence will be generated using random permuted blocks of unequal length. The allocation sequence will be generated by the statistician, blinded to outcome measures and stored in REDCap. Allocation sequence has been derived by the statistician using Stata software. Patients will be randomly assigned to either cefiderocol or best available alternative therapy in a 1:1. Randomisation will be stratified by severity of underlying co-morbidities, as assessed by a Charlson co-morbidity index (≥4 or < 4) and region (Australia, South East Asia, Europe/Turkey).

### Concealment mechanism {16b}

The allocation sequence generated by the trial statistician is stored in REDCap and concealed from users when undertaking randomisation.

### Implementation {16c}

Random sequence will be generated using random permuted blocks of unequal length. The randomisation process will be managed by the University of Queensland via an online module within the REDCap data management system. Patients will be randomly assigned to either cefiderocol or the best available alternative therapy in a 1:1 ratio according to a randomisation list prepared in advance.

## Assignment of interventions: Blinding

### Who will be blinded {17a}

This will be an open-label trial, with the participant, investigator, site study and project management teams being aware of treatment allocation. This includes research staff who will be recording and entering outcome data. Issues that were considered justification for an open-label design included the study’s endpoint (mortality) which is considered a hard endpoint that is not subjective thereby limiting the risk associated with the need to adjust blinded drugs with different pharmacokinetics and dynamics in patients with renal dysfunction. Overall, the open-label trial will provide a population and intervention with greater generalisability, and not compromise internal or external validity.

The trial statistician will be blinded to treatment allocation.

### Procedure for unblinding if needed {17b}

This is an open-label study.

## Data collection and management

### Plans for assessment and collection of outcomes {18a}

A clinical database using the REDCap trial data management system has been developed with a web hosting facility. Electronic case report forms (eCRFs) have been developed and validated to collect all clinical and laboratory-related information. The trial database will include information on demographics (age, gender), underlying illnesses, baseline and follow-up laboratory data including microbiologic data (e.g. organism type, mechanism of resistance and minimal inhibitory concentration (MIC) of study drug), and daily assessments of vital signs and white blood cell counts for the purpose of assessment of clinical outcome. Data on LOS, requirement for ICU admission, duration of ICU admission (if applicable) and discharge destination will also be collected. Source of bacteraemia, if known, as identified by the treating clinicians will be noted. From the data entered into the eCRF, the study team will calculate the scores from the validated scoring systems for outcome data (i.e. SOFA, FBOS). The study team will manage the data and will conduct quality control of the data following their own standard operating procedures.

### Plans to promote participant retention and complete follow-up {18b}

Participants will be hospitalised inpatients with the expected duration of the trial discussed as a component of informed consent. As such there will be direct observation of participants during the trial. Subjects may voluntarily withdraw their consent for study participation at any time and for any reason, without penalty. In all cases, the reasons why a participant is withdrawn must be recorded in detail and entered into the eCRF. Participants may be contacted directly for follow up of secondary endpoints post discharge from hospital.

### Data management {19}

Data for this study will be recorded using REDCap. Data will be stored in a re-identifiable manner in the database using a unique study number for each participant. The database will contain validation ranges to minimise the chance of data entry errors. An audit trail will maintain a record of initial entries and changes made; reasons for change; time and date of entry; and user name of person who made the change. Data queries will be raised by the project manager/delegate and missing data or suspected errors will be raised as data queries and resolved prior to database lock and analysis. The database will contain in-line capability so that these queries and answers are logged as part of the audit trail. Individuals will be trained and issued log-in details and access will be restricted to necessary fields only.

Following each study visit, the designated site staff will complete the visit specific eCRF. Once all required information is received the eCRF shall be considered complete. UQCCR trial staff will then monitor the data for completeness and accuracy. Any eCRF discrepancies, either manual or automatic, will be addressed with the site staff for clarification.

REDCap is held on a specific server at the University of Queensland using standard industry SSL to ensure data privacy as per UQ Cyber Security Policy and Procedures.

These records, electronic and physical, will be kept for a minimum of 15 years after the completion of the trial before being destroyed or erased, as per NHMRC guidelines. These documents will be retained for a longer period if required by the applicable regulatory requirements or institutional policy.

### Confidentiality {27}

All study findings and documents will be regarded as confidential. The investigators and other study personnel must not disclose such information without prior written approval from the Principal Investigator. Subject confidentiality will be strictly maintained to the extent possible under the law and local hospital policy. Identifiable information will be removed from any published data.

### Plans for collection, laboratory evaluation and storage of biological specimens for genetic or molecular analysis in this trial/future use {33}

Blood cultures and other blood laboratory tests will be collected as per local clinical procedures, using standard blood culture bottles and recommended blood volumes, an EDTA tube (4–6 ml) for FBC, and a lithium heparin tube (4–6 ml) for LFTs, EUC and CRP. The clinical team will arrange these tests as part of usual care but the research team will ensure blood is collected and analysed. This testing will be performed as per the trial schedule. The study will collect routine laboratory data and enter it into the eCRF.

All blood cultures which flag positive will be processed as per the local laboratory’s usual procedures. Microbiology laboratories at study sites will perform susceptibility testing for cefiderocol for each entry blood culture isolate as per the laboratory manual for the trial. All bacterial isolates will be frozen and stored as per standard laboratory practice at each site. Bacterial isolates collected from blood cultures will be shipped to the Centre for Clinical Research, University of Queensland for confirmatory susceptibility testing and genetic analysis for potential mechanisms of resistance. An aliquot of the initial blood culture isolate (as a suspension of pure bacterial colonies) will be stored at -80^o^C in glycerol and nutrient broth (e.g. TSB) at the local laboratory and shipped in batches to Australia. The timing of which will be determined by the rate of recruitment at the site and liaison between the local laboratory and coordinating centre at UQCCR.

## Statistical methods

### Statistical methods for primary and secondary outcomes {20a}

A modified intention-to-treat (mITT) analysis approach, will be adopted to make inference on the possible non-inferiority of the treatment arm, compared to the control arm, in terms of 14-day mortality. Absolute difference in mortality at 14 days and a 95% CI will be calculated. Further details will be provided in a detailed Statistical Analysis Plan.

If the primary objective meets the criteria for non-inferiority for the study drug (cefiderocol) compared with the control arm, a secondary analysis examining for superiority will be undertaken.

### Methods for additional analyses (e.g. subgroup analyses) {20b}

The primary outcome analysis will be undertaken in the following sub-groups: (1) urinary versus non-urinary source; (2) Carbapenem non-susceptible pathogens vs carbapenem susceptible pathogens; (3) Acinetobacter spp vs non-Acinetobacter spp.; and (4) Charlson co-morbidity index ≥ 4 vs Charlson co-morbidity index < 4. Heterogeneity of treatment effect (on the odds ratio scale) will be explored across sub-groups using a test for the intervention × subgroup interaction by adding this term and the subgroup as covariates in a logistic regression model.

### Methods in analysis to handle protocol non-adherence and any statistical methods to handle missing data {20c}

Study participants who receive at least one dose of the study drug will be included in the modified intention-to-treat (mITT) population. Participants who receive at least 5 days of study drug will be included in the per-protocol (PP) population. The primary outcome will be determined for the mITT and PP populations.

For every day where a numerical value is missing, the last measured value will be carried forward for each day until either a new value is recorded, the patient withdraws from the study or the patient dies, unless the outcome has already been reached. Patients who fail to have a day 3 blood culture collected but were otherwise afebrile (temp < 38 °C) will be assumed to have achieved microbiological resolution (i.e. negative blood cultures). Otherwise, no imputation of missing data will be conducted. Missing data for relevant study parameters, if any, will be presented in accordance with standard procedures and compared across study arms.

## Oversight and monitoring

### Composition of the coordinating centre and trial steering committee {5d}

The University of Queensland is the study sponsor with the steering committee comprising staff from the University of Queensland Centre for Clinical Research. This includes the Principal investigator, the study project manager, co-investigator and trial statistician.

### Composition of the data monitoring committee, its role and reporting structure {21a}

A DSMB will be established, comprising two independent infectious disease physicians and an independent statistician. The DSMB may be provided with details of outcomes according to the treatment arm. The interim analysis will be communicated to the local trial team as well as all national and international collaborators along with the DSMB recommendations for action. If there is a significant safety concern raised, the DSMB may recommend to the Principal Investigator that the trial should be stopped with the final decision on trial termination to be the responsibility of the trial steering committee. The DSMB will recommend if the study should continue or be stopped given the results of the primary efficacy endpoint of mortality at day 14 at a level of significance of *P* < 0.001 (Peto rule) and is it safe for the study to continue with regard to serious adverse events (SAEs)?

Additional interim data summaries may be considered by the DSMB.

### Interim analyses {21b}

As requested by an ethics committee, a limited interim analysis will be performed after the first 10 and 50 subjects have completed the 14-day study period. Further interim analyses may be requested by the DSMB.

### Adverse event reporting and harms {22}

Adverse events (AE) are assessed in accordance with the definitions in the International Conference on Harmonisation (ICH) Guideline for Good Clinical Practice which includes the delineation of serious adverse events (SAE). Events will be reviewed and classified by the site principal investigator. The relationship of the event to the study drug will be assessed based on whether the event can be reasonably explained that the study treatment caused the event or not.

The treating team has the primary responsibility for reviewing laboratory test results and determining whether an abnormal value in an individual study participant requires action. In general, abnormal laboratory without clinical significance (based on clinical judgement) should not be recorded as adverse events; however, laboratory value changes requiring therapy or adjustment in prior therapy are considered adverse events. The investigators should liaise closely with the treating teams and remain aware of any such adverse events.

As this study involves critically or severely ill patients, it is anticipated and expected that many participants will experience events that might be considered AEs or SAEs, but are expected features of critical illness requiring intensive care.

Adverse events will be classified by system organ class and preferred term using Medical Dictionary for Regulatory Activities (MedDRA).

AE’s will be reported from the first study dose through to 5 days after the final dose of study medication. All Serious Adverse Events (including SUSARs) will be reported to UQCCR within 24 h of the site being made aware. Standardised reporting forms for SAEs will be provided to all sites (sites are able to use locally available SAE templates, with prior approval from the UQCCR project team). The investigator must also comply with all applicable ethical and regulatory requirement/s relating to the reporting of serious adverse events.

Pre-existing conditions or diseases that occur during the study (e.g. seasonal allergies, asthma or recurrent headaches) should not be considered as adverse events unless they change in frequency or severity. AEs include any occurrences that are new in onset or aggravated in severity or frequency from the baseline condition, or abnormal results of diagnostic procedures, including laboratory test abnormalities. Lack of efficacy, aggravation or relapse of current infection is not an (S)AE in the study.

Management of adverse events, including the decision to cease study drug, will be at the discretion of the treatment team in discussion with the research team.

### Frequency and plans for auditing trial conduct {23}

Specific monitoring will be conducted by clinical trial monitors, or designees, who will perform monitoring activities in accordance with the study monitoring plan. This will involve on-site visits and remote monitoring activities as necessary including; site file review, review of Informed Consent Forms, Source Data Verification (SDV) and Serious Adverse Event (SAE) review as per the study monitoring plan.

If it is not possible for representatives of the sponsor to perform on site monitoring visits due to travel restrictions or entry restrictions into hospitals due to COVID-19, key data fields (efficacy and safety data points) will be remotely monitored at UQCCR.

Audits may be conducted by regulatory authorities.

### Plans for communicating important protocol amendments to relevant parties (e.g. trial participants, ethical committees) {25}

The trial steering committee will communicate with all study sites and relevant ethics committee with regards to protocol changes or other study changes or management.

### Dissemination plans {31a}

The results will be submitted to ClinicalTrials.gov, presented at national and international conferences, and be submitted for publication with a peer-reviewed journal(s).

## Discussion

Bloodstream infections from Gram-negative bacteria are associated with a mortality rate of > 10% [13, 14]. In the presence of carbapenem resistance, case series have shown that can exceed 40% of cases [15, 16]. Increasing carbapenem use in the face of antimicrobial resistance is a potential driver for carbapenem resistance is of significant concern requiring the consideration of carbapenem sparing options [17]. Hence there is a need for the development of new antibiotics with broad activity against Gram-negative pathogens such as cefiderocol. While data exists for this agent in other settings (complicated urinary tract infection and hospital-acquired pneumonia), the main objective of the study is to examine cefiderocol in an area of significant clinical need, that of bloodstream infections and sepsis. By selecting a population at risk for multi-drug-resistant pathogens and commencing study treatment early in the clinical illness (within 48 h of index blood culture) the trial hopes to provide guidance to clinicians about the role of cefiderocol in this setting.

## Trial status

To date, ethical and regulatory approval has been obtained for sites in Australia, Singapore and Thailand. Sites in Australia, Singapore and Thailand have been activated to commence recruitment, with the first patient recruited in December 2019. On the date of submission, the current version of the protocol was version 7.0, protocol date 25th May 2020. Recruitment is planned to continue until late 2022.

## Data Availability

The owner of the data is the sponsor, the University of Queensland. Data related to adverse events will be shared with Shionogi Pharmaceutical as per the pharmacovigilance agreement between the two parties. The data cannot be used or disclosed to a third party without prior permission.

## Abbreviations

AE: Adverse event
ALT: Alanine aminotransferase
AST: Aspartate aminostransferase
CDI: *Clostridium difficile* infection
CRAB: Carbapenem-resistant *Acinetobacter baumanii*
CrCl: Creatinine clearance
COVID: Coronavirus disease
CRE: Carbapenem-resistant Enterobacterales
CRP: C-reactive protein
CVVH: Continuous veno-venous haemofiltration
CVVHD: Continuous veno-venous haemodialysis
CVVHDF: Continuous veno-venous haemodiafiltration
DSMB: Data and Safety Monitoring Board
eCRF: Electronic case report form
EDTA: Ethylenediamine tetra-acetic acid
ESBL: Extended-spectrum beta-lactamase
ESRD: End-stage renal disease
EUC: Electrolytes, urea and creatinine
FBC: Full blood count
FBOS: Functional Bacteraemia Outcome Score
FDA: Food and Drug Administration
GCP: Good Clinical Practice
HREC: Human Research Ethics Committee
IAI: Intra-abdominal infection
IB: Investigator’s Brochure
ICH: International Conference on Harmonisation
ICU: Intensive care unit
IRB: Institutional Research Board
ISF: Investigator Site File
IV: Intravenous
LOS: Length of stay
MDR: Mult-drug-resistant
mITT: Modified intention to treat
MRO: Multi-resistant organism
MRSA: Methicillin-resistant *Staphylococcus aureus*
PI: Principal Investigator
PICF: Participant information sheet/consent form
PP: Per protocol
RCT: Randomised controlled trial
REDCap: Research Electronic Data Capture
SAE: Serious adverse event
SDM: Substitute decision-maker
SDV: Source document verification
SOC: Standard of care
SOFA: Sequential Organ Failure Assessment Score
SOP: Standard operating procedure
SUSAR: Suspected unexpected serious adverse reaction
UQ: University of Queensland
UQCCR: University of Queensland Centre for Clinical Research
VRE: Vancomycin-resistant Enterococci
